# Quality Control of Biomedicinal Allergen Products – Highly Complex Isoallergen Composition Challenges Standard MS Database Search and Requires Manual Data Analyses

**DOI:** 10.1371/journal.pone.0142404

**Published:** 2015-11-11

**Authors:** Jelena Spiric, Anna M. Engin, Michael Karas, Andreas Reuter

**Affiliations:** 1 Division of Allergology, Paul-Ehrlich-Institut, Langen, Germany; 2 Institute of Pharmaceutical Chemistry, University of Frankfurt, Frankfurt, Germany; Moffitt Cancer Center, UNITED STATES

## Abstract

Allergy against birch pollen is among the most common causes of spring pollinosis in Europe and is diagnosed and treated using extracts from natural sources. Quality control is crucial for safe and effective diagnosis and treatment. However, current methods are very difficult to standardize and do not address individual allergen or isoallergen composition. MS provides information regarding selected proteins or the entire proteome and could overcome the aforementioned limitations. We studied the proteome of birch pollen, focusing on allergens and isoallergens, to clarify which of the 93 published sequence variants of the major allergen, Bet v 1, are expressed as proteins within one source material in parallel. The unexpectedly complex Bet v 1 isoallergen composition required manual data interpretation and a specific design of databases, as current database search engines fail to unambiguously assign spectra to highly homologous, partially identical proteins. We identified 47 non-allergenic proteins and all 5 known birch pollen allergens, and unambiguously proved the existence of 18 Bet v 1 isoallergens and variants by manual data analysis. This highly complex isoallergen composition raises questions whether isoallergens can be ignored or must be included for the quality control of allergen products, and which data analysis strategies are to be applied.

## Introduction

Allergen products that are used to diagnose or treat allergic diseases are defined as “Pharmaceutical preparations derived from extracts of naturally occurring source materials containing allergens…” [1]. They often contain more than one allergen and an unknown number of non-allergenic proteins. Additionally, it is well known that certain allergens comprise a large number of closely related sequence variants so-called isoallergens (>67% of sequence identity) or variants (>90% sequence identity) [2]. The diagnostic or therapeutic performance of such products is largely determined by the quality and composition of the initial protein extract. Regulatory authorities and pharmaceutical industry apply several analytical methods to characterize or standardize such products. One of the most important parameters is the IgE-antibody binding capacity, i.e., potency. Allergen specific IgE antibodies are induced in predisposed individuals in course of the development of the Type I allergy. It is generally accepted that the binding of the allergen products to IgE and/or IgG antibodies indicates the presence of allergens, and that the intensity allows for a quantitative evaluation of the total allergen content and of the quality of the product [3]. Currently available potency assays do not differentiate between: 1) clinically relevant and irrelevant allergens; 2) products with the same potency but different individual allergen content; 3) parameters which have an impact on IgE binding, e.g., protein folding; 4) closely related sequence variants, i.e., isoallergens, even though isoallergens are considered to be clinically relevant [4]. Other assays are applied to quantify individual allergens (e.g.,ELISA) or to record protein profiles (e.g.,SDS-PAGE). However, the shortcomings of ELISA are well known and mainly related to the antibodies used. Most importantly, ELISA may not pick up certain isoallergens at all and may give an incorrect quantitative readout [5]. Additionally, ELISA do not generally allow quantifying different isoallergens in mixtures, even though Foetisch and co-workers have shown that this is possible for two isoallergens of the major carrot allergen, Dau c 1 [6]. However, Dau c 1.01 and Dau c 1.02 are fairly different and share only 50% of the amino acid sequence while isoallergens or allergen variants share more than 67% or more than 90% of the amino acid sequence, respectively. Mass spectrometry has been applied for allergen identification since the late 90s [7] as well as to identify isoallergens [8–10], whilequantitative information was provided approximately ten years later [11]. Most of the studies rather aimed on identifying novel isoallergens than on evaluating the entire isoallergen composition systematically.

Birch pollen, as one of the most important allergen sources in central Europe [12], contains five known allergens, Bet v 1 [13], Bet v 2 [14], Bet v 4 [15], Bet v 6 [16, 17] and Bet v 7 [18] and Bet v 1 alone exhibits a substantial degree of heterogeneity. Several pathogenesis-related genes have been identified to encode a mixture of Bet v 1 isoallergens and variants [19–21]. Only five studies were published aiming at determining the isoallergen composition of Bet v 1 at the protein level [10, 20–23]. There are two main conclusions which can be drawn from these studies. First, the experimental setup and methodological details do not allow to unambiguously prove the existence of isoallergens and variants in a comprehensive way. Second, there is a striking mismatch between the total number of Bet v 1 isoallergens being reported at the nucleic acid level and the number that was found at the protein level. The UniProt database contains more than 90 entries for *Betula Pendula*, that were mostly deduced from nucleic acid sequences, but only between 3 and 9 different Bet v 1 isoallergens were found to co-exist at the protein level by several research groups when an individual pollen preparation was under investigation. Swoboda and co-workers [21] used a monoclonal antibody to purify Bet v 1 prior to MS analysis, and they might have lost a certain part of the Bet v 1 composition. Bollen et al. [22] precipitated the proteins before applying a two-step chromatographic purification procedure taking the same risk. In most cases the isoallergens were either in-gel digested after SDS PAGE [20–23] or digested in solution [22]. Either procedure leads to a mixture of peptides which cannot easily be re-assembled to a specific protein, if a mixture of isoallergens with multiple peptides representing the same amino acid stretch is under investigation. This issue is well known as the”protein inference problem” and especially hampers the data analysis of shotgun experiments [24]. A large number of studies aimed on developing algorithms to improve the assembly of identified peptides to proteins [24–26]. However, all software tools available to date only improve the assembly and reduce the false discovery rate rather than allowing absolutely unambiguous identification of sequence variants [26–29]. A more recent study published in 2011 [10] addressed this problem by determining the intact mass of Bet v 1 after 2-D separation. However, the overlapping molecular ion signals and isotopic clusters of Bet v 1 isoallergens and variants hamper the intact mass analysis if the most common and frequently observed modifications, such as deamidation of glutamin and asparagin and oxidation of methionine are considered. The latter study reported only 5 isoallergens. Even though it remains doubtful whether MS applied on samples that contain several sequence variants allows one to unambiguously assign intact masses to specific sequences, we do believe that Erler et al [10] very likely missed some isoallergens which had an overlapping mass rather than reporting false positive hits.

Referring to these current limitations we focused on two major aims: 1) Development of an experimental setup, including a suitable manual data analysis strategy, which allows one to unambiguously identify closely related sequence variants; 2) Comprehensive and reliable evaluation of the allergen composition of birch pollen with special emphasis on the Bet v 1 isoallergen composition.

## Materials and Methods

All chemicals and reagents were of analytical grade, unless specified otherwise.

### Sample preparation

Birch pollen (*Betula Pendula*) was purchased from Allergon (Allergon AB, Ängelholm, Sweden). Proteins were extracted from birch pollen with Tris-Borate buffer (8mM Tris with 10mM (NH_4_)_2_B_10_O_16_; pH8.5; in 1/10 w/v ratio) for 6h at 4°C at 500 rpm (Reax 2, Heidolph Instruments Schwabach, Germany), followed by centrifugation at 16 000 g, and filtration using a 0.22μm nitro cellulose membrane (Sartorius AG, Goettingen, Germany). The protein concentration was determined by the Bradford assay using bovine serum albumin as standard. The extract was stored at -80°C.

### Two-dimensional electrophoresis

Two dimensional electrophoresis was performed according to Goerg et al. [30] and modified as described below. Fifty microgram of birch pollen proteins were diluted in 200 μl rehydration buffer (7 M Urea, 2 M Thiourea, 4% CHAPS, 20 mM Tris, 65 mM DTT, and 0.2% Biolyte 3–10 or 7–10). Isoelectric focusing (IEF) was carried out in PROTEAN®IEF Cell using ReadyStrip IPG strips 11 cm, pH3-10, pH 3–6, 4–7, 5–8, or 7–10 (Bio-Rad laboratories, Hercules, USA). After 12 h of active rehydration at 50 V, proteins were focused at 150 V, 500 V; and 1 200 V for one hour each, at 4 000 V for 2 h and at 8 000 V until 30 000 Vhrs were reached. Additionally 1 000 V were applied until the run was manually stopped. After IEF, the IPG strips were reduced in equilibration buffer (6 M Urea, 0.375 M Tris-HCl, 2% SDS, 20% Glycerol, pH 8.8) containing 65 mM DTT for 15 min, and alkylated for another 15 min in the same buffer having 260 mM Iodoacetamide at room temperature. Second dimension separation was performed using a Criterion cell, and proteins were separated on a 12% Criterion™ XT Bis-Tris polyacrylamide gel, 13.3 x 8.7 cm, (Bio-Rad) using NuPAGE® MOPS-SDS as a running buffer (Novex by Life Technologies, Grand Island, USA) at constant voltage of 200 V for 50 min. Low molecular weight marker (Amersham, GE Healthcafe, UK) was used for molecular weight determination, and the proteins were stained with SyproRuby (Invitrogen by Life Technologies) according to the manufacturer’s instructions. The images were captured using a laser scanner (FLA-9000 FUJIFILM Corporation, Japan). The spots were excised and subjected to MS analysis.

### In-gel protein digestion

In-gel digestion was performed according to Shevchenko and co-workers [31] and modified as specified below. Protein spots were washed four times for 15 min in destaining solution (40% Ethanol/50 mM NH_4_HCO_3_), reduced with 65 mM DTT in 50 mM NH_4_HCO_3_ for 20 min at 600 rpm, and alkylated with 260 mM Iodoacetamide in 50 mM NH_4_HCO_3_ for 20 min at 600 rpm using thermomixer (Eppendorf, Hamburg, Germany). The gel plugs were dehydrated with 100% acetonitrile (ACN), and vacuum dried using Savant Speed Vac® (Thermo Fisher Scientific, Schwerte, Germany), followed by rehydration in 25 mM NH_4_HCO_3_ containing 75 ng/μl of trypsin (Trypsin from porcine pancreas, Proteomics Grade, Sigma-Aldrich, St. Louis, USA). Digestion was carried out for 3 h at 37°C and continued over night after addition of elution buffer (25 mM NH_4_HCO_3_/10%ACN), in thermal cycler (Techne by Bibby Scientific, Staffordshire, UK). The digestion was stopped by addition of 5% formic acid (FA) to a volume of 10% of the total volume. The samples were stored at -80°C until further analysis.

### LC-MS^E^ analysis

Samples were analyzed using a nano-UPLC ESI Q-TOF-MS (nanoACQUITY UPLC and Synapt MS, Waters Milford, USA). A total volume of 8 μl was injected, desalted on-line at a flow rate of 5 μl/min 99% solvent A (H2O + 0.1%FA) and 1% solvent B (ACN + 0.1% FA) using a trap column (nanoACQUITY Trap C18, 5 μm, 180 μm X 20 mm, Waters). The peptides were separated using an analytical column (nanoACQUITY C18, 1.7 μm, 100 μm X 100 mm; Waters) at a flow rate of 0.5 μl/min, with 97% A for 1 min, a linear gradient to 60% A for 30 min, 95% B for 1 min, followed by 97% A for 18 min. A potential sample carryover was monitored by analyzing 100 fmol of Enolase standard solution (Waters) between sample runs. The MS was operated in positive V-mode using standard parameters and lockmass calibrated (Glu-1-Fibrinopeptide 1 pmol/μl, at 0.5 μl/min, 1 scan every 20 sec). Data were acquired using data-independent acquisition (MS^E^) mode, altering between low (4 V) and high (ramped from 15 V-30 V) collision energies, with a scan time of 0.4 sec. The data were acquired in *m/z* range from 50 to 1990.

### Data processing and database searches

The MS^E^ data were processed using ProteinLynx Global Server (PLGS) version 2.4 (Waters) applying the parameters indicated below. Chromatographic peak width and MS-TOF resolution were set to automatic, lock mass for charge 1+ was 684.3469, for charge 2+ 785.8426, a 2.5 Da lock mass window was used, low and elevated energy thresholds were set to 250 or 100 counts, respectively, intensity threshold was 1500. For initial protein identification, all data files were searched against UniProt database restricted to green plants (*Viridiplantae*) as from march 2012. Additionally, an in-house-database consisting of Uniprot database restricted to Bet v 1 isoallergens from *Betula Pendula* was created for identification of Bet v 1 isoallergens. Fifteen redundant (100% sequence identity to P15949) sequences were removed from this database. The database search parameters were: a maximum of one missed cleavage site, a minimum of 3 fragment ion matches per peptide, a minimum of 7 fragment matches per protein, and a minimum of 1 peptide match per protein with a set false positive rate of 4%. Peptide and fragment tolerance was set to automatic. Fixed modifications were restricted to carbamidomethyl of Cys, and variable modifications to deamidation of Asn and Gln and oxidation of Met.

## Results and Discussion

### 2D-PAGE of birch pollen and basic identification of proteins

**Fig 1:**
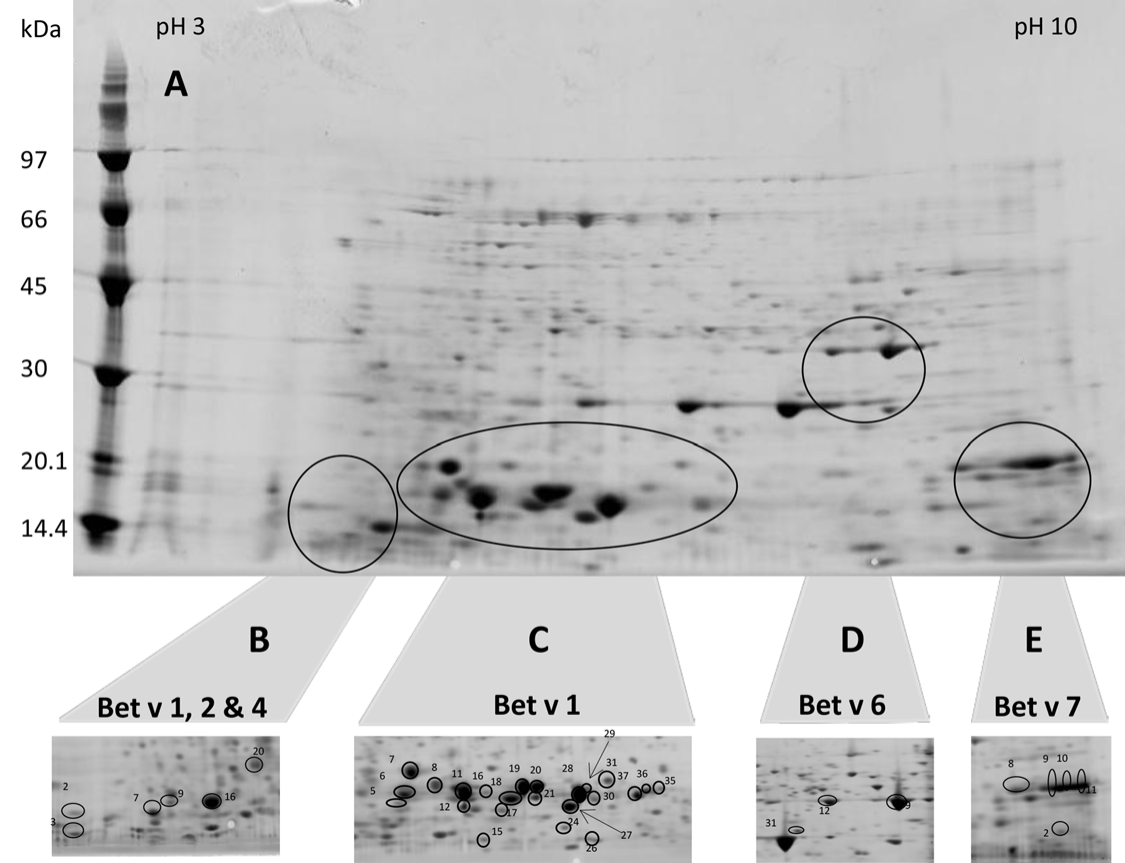
2-D maps of birch pollen extract. [A] pH 3–10 entire protein profile; Panels B-E show cropped images focusing on zones containing allergens. [B] pH 3–6 pH; [C] pH 4–7 pH; [D] pH 5–8; [E] pH 3–10.

Fig 1 shows a 2D-PAGE gel of the total proteome of birch pollen between pH 3 and pH 10. Numerous proteins were identified including all known birch pollen allergens. However, the separation of Bet v 1 related proteins was not sufficient to allow an unambiguous identification of isoallergens, as too many isoallergens and variants were co-migrating. We further improved the resolution by using narrow-range pH-strips to zoom into those parts of the proteome where Bet v 1 (Fig 1, panel C), Bet v 1, 2 & 4 (panel B), and Bet v 6 (panel D) were found. Bet v 7 (panel E) was identified using pH3-10 strips. We studied 178 spots of which 74 gave a positive result. The majority of negative spots were of low intensity. Nevertheless, the identification of some fairly intense spots failed very likely due to a lack of sequences in the UniProt database, as the genome of birch pollen is not yet fully sequenced and published. Only spots containing allergens are marked in panel B–E. Full-size versions of these images including a collection of all other spots with positive protein identification are supplied as supporting Information (Figure A in S1 File, Panels A-F). In total 47 different non-allergenic proteins were identified within 42 spots; detailed information is provided in supporting information (Tables A–D in S1 File; Table A in S1 File). Details on the identification of the birch pollen allergens Bet v 2, 4, 6 and 7 can be taken from Tables A- D in S1 File; Table B in S1 File. Briefly, Bet v 2 was found in 4 spots (Fig 1, panel B, no. 2, 7, 9 & 16), Bet v 4 in one spot only (panel B, no. 3), Bet v 6 in 3 spots (panel D, no. 9, 12 & 31) and Bet v 7 in 5 spots (panel E, no. 2, 8, 9, 10 & 11). The number of identified peptides ranged from 1–23, the PLGS score from 291–35 265 and the mass error from 1.6–8.1 ppm. We found no evidence for sequence variants of Bet v 2, 4, 6 or 7. Even though we did not specifically focus on this, we assume that the occurrence of multiple spots containing these allergens was caused by post translational or artificial modifications. The results of the basic protein identification are summarized in Table 1.

**Table 1:** Summary of proteins identified on 2D-Gels.

Protein Type	Allergen	Spots	Isoallergens and variants
PR 10 protein	Bet v 1	24	up to 7
Profilin	Bet v 2	4	1
Polcalcin	Bet v 4	1	1
Phenylcoumaran benzylic ether reductases	Bet v 6	3	1
Peptidyl prolyl cis trans isomerase	Bet v 7	3	1
Non-allergenic proteins	-	42	-

Proteins are represented by protein type, allergen name, number of spots found and number of isoallergens and variants found per spot.

### Identification of isoallergens and variants of Bet v 1 by manual data interpretation

The analysis of the MS data for Bet v 1 spots was difficult and required extensive manual data interpretation. The result of an initial data analysis was that Bet v 1 related sequences were present in 24 different spots. This agreed to previous publications that individual spots contain multiple sequence variants of Bet v 1. However, the automatic output of PLGS data base search using the UniProt database restricted to green plants did not allow determining the detailed type and number of isoallergens or variants. As an example, for spot no. 11 (Fig 1 panel C) PLGS reported one “hit” comprising more than 100 individual proteins. We were tempted to conclude that this indicates the presence of one sequence variant only. A short glance at the details revealed the presence of several different spectra corresponding to the same region of Bet v 1 and clearly indicating distinct sequence variants. However, it was also very obvious that some of the sequences were 100% identical and others showed amino acid sequence variation for which we could not find experimental evidence. These proteins were grouped into one hit very likely representing the six different basic peptide grouping scenarios reported by Nesvizhskii and Aebersold [24].

To elucidate the real Bet v 1 isoallergen composition, we established a special workflow which included a two-step database search combined with a very conservative manual assignment of peptide sequences to selected database entries. Initially, a standard database search provided the basic identity of a protein supported by score, sequence coverage, average mass error, number of peptides and other parameters. This allows an unbiased conclusion about the basic identity of a protein. All results indicating proteins other than Bet v 1 were reported without further manual selection and are presented in Tables A–D in S1 File; Table A in S1 File (non-allergenic proteins) or Tables A–D in S1 File; table B in S1 File (birch pollen allergens other than Bet v 1). A second targeted database search was performed for those spots that contained Bet v 1 related sequences. This database was restricted to Bet v 1 sequences of *Betula Pendula*, and this restriction lowered the number of database hits down to a level that allowed a manual data interpretation. We manually extracted two pieces of information from this database search: 1) a list of protein entries suggested by PLGS; 2) a list of all different peptides that were used to identify the above mentioned protein entries. The next step aimed on improving the quality of this data set. The quality, i.e., the score of pass-one matches, is considered to be high enough to identify a protein on its own, whereas spectra of pass-two matches are only used to provide additional information on already identified proteins, e.g., to improve sequence coverage. Consequently, we removed all pass-two matches from the list of peptides. The manual assignment of peptides to protein entries was started by selecting the protein entry that was identified with the largest number of individual peptides. This matched, in most cases, the protein identified from PLGS with the highest sequence coverage. Next, all those protein entries that were identified using the same set or a fraction of these peptides were manually removed from the list. Likewise, all those peptides that were used to identify the first protein entry were removed. This process was repeated with the remaining proteins entries and the remaining peptides as often as required until all peptide sequences were assigned to a protein entry. This iterative procedure did not essentially provide novel or additional information as compared to the automatic output of PLGS, but it made the information concerning sequence variants accessible and utilizable by removing large amounts of redundant information. This procedure clearly improved the quality, i.e., the reliability of the results. PLGS often indicated the presence of a Bet v 1 variant on the basis of a certain number of peptides that were shared by many sequence variants and on the basis of only one peptide that was specific for this particular Bet v 1 variant. In some cases these variant-specific peptides were classified as pass-two matches, i.e., to be of an arguable quality. This means that this particular sequence variant was essentially identified on the basis of one single spectrum only, but the total protein score also included peptide scores of all other commonly shared peptides. This raises doubts whether the total protein score, sequence coverage and average mass error are sufficient to give evidence concerning sequence variants. A statistically significant score may suggest a level of reliability that is not fully supported by the experimental data. We decided to remove 4 additional Bet v 1 isoallergens from our results list, because each of these protein entries was suggested by PLGS on the basis of one specific spectrum of doubtful reliability.

**Fig 2:**
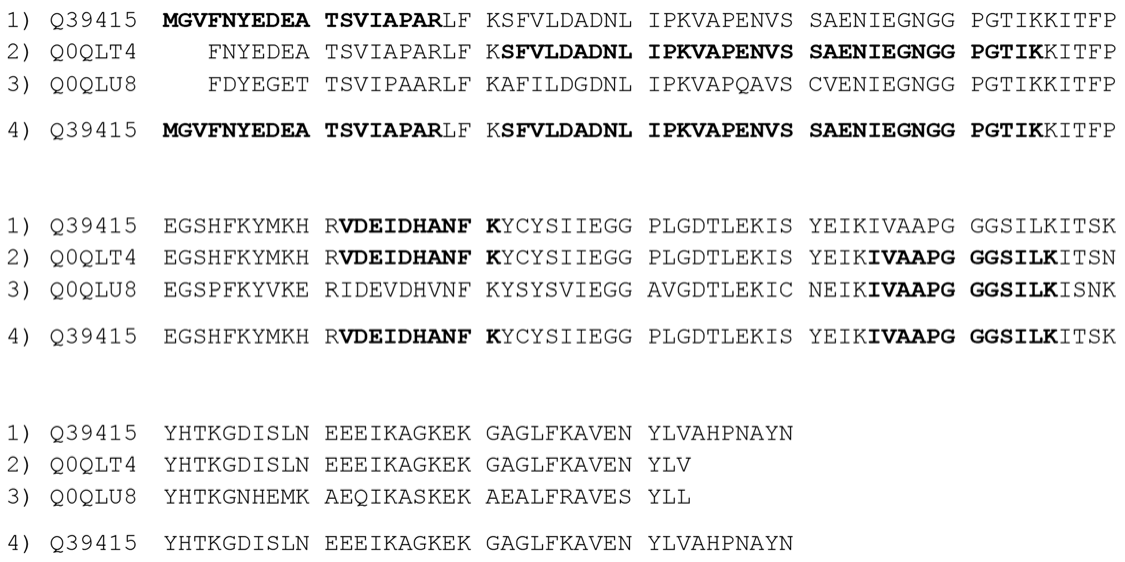
Apparent random assignment of peptides to protein entries. Peptides that were identified by MS database search are printed in bold. Lines 1, 2 and 3 show the results of the automatic assignment of PLGS. Line 4 shows the result of manual assignment.

Another issue that was revealed by manual data analysis was an apparently random assignment by PLGS of spectra to several different protein entries that could have been assigned to one protein entry. This phenomenon is illustrated in Fig 2 using experimental data of spot 28 (Fig 1, panel C) and represents a new peptide grouping scenario that was not reported by Nesvizhskii and Aebersold [24]. PLGS reported 5 peptide sequences being identified with MS database search. The peptide representing the N-terminal part of the protein was correctly assigned only to entry Q39415 as the N-terminal parts of other entries were incomplete. The successive peptides (SFVL… and VAPEN…) were assigned to Q0QLT4 only even though they also match Q39415. Another peptide (VDEI…) was correctly assigned to Q39415 and Q0QLT4 only. The fifth peptide was finally assigned to Q0QLT4, even though the third entry, Q0QLU8, and Q39415 would match, too. Manual assignment of the same peptide sequences leads to one protein entry only as all peptides match to Q39415. The protein entries Q0QLT4 and Q0QLU8 are essentially not needed to explain the experimental data following the principle of Occam’s razor [32]. Taken together, our iterative manual procedure allowed explaining the experimental MS data with the lowest possible number of protein entries, rather than reporting either redundant sequences or even false positive hits.

### Summary of the identification of Bet v 1 isoallergens and variants

**Fig 3:**
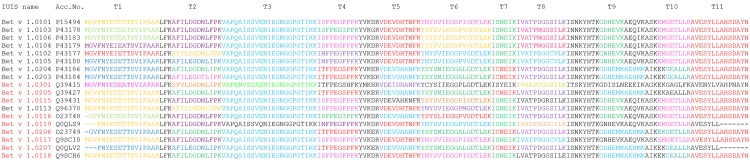
Sequence alignment of all 18 Bet v 1 isoallergens and variants that were unambiguously identified with MS. The allergen names printed in black were previously approved by the IUIS Allergen Nomenclature Subcommittee. The names printed in were IUIS approved on the basis of this study. All amino acid residues printed in black were not detected with MS. T1-T11 classifies the sequence stretches of different tryptic peptides of Bet v 1. Peptides with identical colors within a sequence stretch have an identical amino acid sequence.

The Bet v 1 isoallergens that were identified applying this iterative process are summarized in Fig 3. In total we reliably detected 18 different sequences in our birch pollen extract with sequence coverages between 47% and 91%. These sequences are represented by 3 different isoallergens. We found 12 variants of Bet v 1.01, five variants of Bet v 1.02, and one variant of Bet v 1.03. Moreover, we detected between 1 and 7 isoallergens and variants within one spot. This agrees with previous publications discussing that posttranslational or chemical modifications may lead to multiple spots from one protein [9, 33, 34]. However, Bet v 1.0203, 1.0401, 1.0115, 1.0301, and1.0118 were detected in one spot only. It is very interesting to note that not a single peptide is shared by all Bet v 1 entries, while tryptic peptides T3 and T11 are shared by 16 or 17 entries, respectively. The peptide T3 is different in Bet v 1.0301 and Bet v 1.0119 whereas the peptide T11 is different in Bet v 1.0301. We assume that Bet v 1.0119 and Bet v 1.0207 share the T11 peptide even though the database entries are incomplete. All other tryptic peptides are characterized by a fairly large degree of heterogeneity. However, the vast majority of these peptides are still shared between many entries in varying combinations. Only 8 entries (Bet v 1.0104–T1, Bet v 1.0102-T1, Bet v 1.0203-T2, Bet v 1.0301-T1-T2-T3-T5-T6, Bet v 1.0115-T6, Bet v 1.0113-T6, Bet v 1.0116-T6 and Bet v 1.0119-T8) exhibit an isoallergen or variant-specific peptide. A detailed summary of the identification of these isoallergens and variants is given in Tables A–D in S1 File; Tables C and D in S1 File. This summary includes standard information such as gel, spot number, accession number, sequence coverage and average precursor error (Tables A–D in S1 File; Table C in S1 File), but also fragmentation spectra (Tables A—D in S1 File; Table D in S1 File) for specific peptides whenever an isoallergen or variant was essentially identified on the basis of only one peptide. Column P* in Tables A–D in S1 File; Table C in S1 File specifies the corresponding peptide sequence for all Bet v 1 isoallergens that were identified on the basis of only a single peptide. For example, isoallergen P43177 was identified in spot 20, gel B on the basis of the peptide T2 with the amino acid sequence AFILDGDNLVPK. All other peptides assigned to this isoallergen were also present in Q9SCI0, Q39427 or 43180. The latter peptides may as well originate from all 4 isoallergens. The only peptide reliably pointing towards the presence of P43177 was T2-AFILDGDNLVPK. This means that P43177 was essentially identified on the basis of only a single peptide, even though 5 more peptides matched the protein sequence. We accepted identifications on the basis of one peptide after manual evaluation of the corresponding fragmentation spectrum.

## Conclusions

Our data have clearly shown that the Bet v 1 isoallergen and variant composition is much more complex than previously suggested. Considering the entire scientific literature and public sequence databases, our findings cannot completely bridge the gap between the huge number of sequences being reported at the DNA level and the limited number of isoallergens that were confirmed at the protein level. We found 18 different sequences being expressed as proteins as compared to only 5 [10], 4 [20], and 7 [21] and app. 9 [23]. However, our findings match fairly well with results of a study specifically aiming on evaluating the Bet v 1 composition of three *Betula pendula* cultivars at the genomic level [19]. The authors reported 44 different nucleotide sequences, assigned to 13 putative genes encoding 14 different sequence variants of Bet v 1, and this is well in line with our findings, considering that we studied an unknown very likely different cultivar. We assume that unintentionally many different cultivars were studied within the last 20 years leading to this huge number of different Bet v 1 sequences at the DNA level.

**Fig 4:**
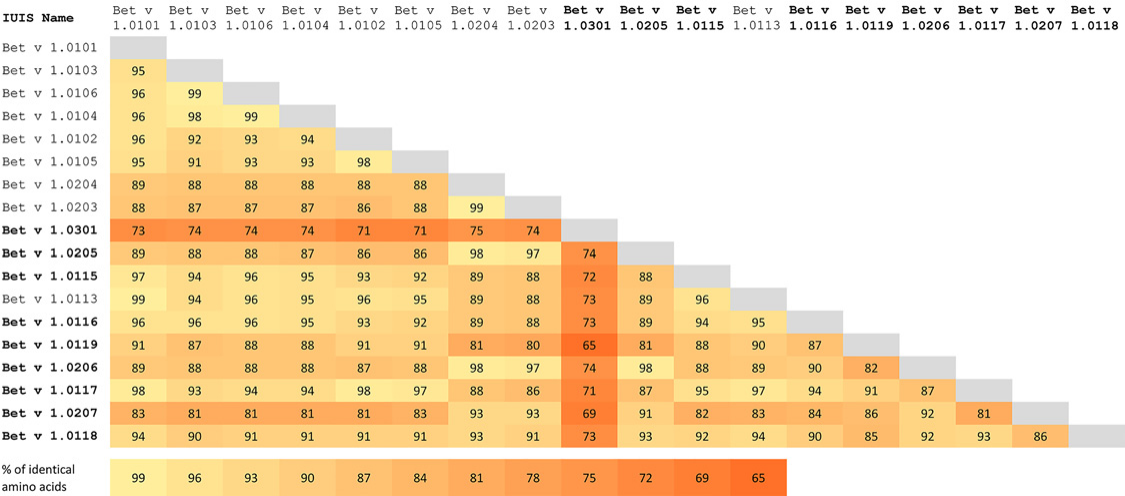
Sequence identities of Bet v 1 isoallergens. Bold letters indicate isoallergens and variants that were IUIS approved based on our study.

However, a comprehensive and conclusive knowledge about isoallergens and variants is very important as it was demonstrated that isoallergens are clinically relevant and show different IgE-antibody binding capacity and different T-cell stimulation properties [4]. A different IgE-binding capacity would have an impact on quality control and standardization involving IgE-based potency assays as well as on the diagnostic performance of test allergens. Varying T-cell reactivity could alter the therapeutic performance, as the specific immunotherapy of type 1 allergy is T-cell driven. We confirmed the presence of one Bet v 1 isoallergen (Bet v 1.0301) not detected at the protein level before; thus, not IUIS approved prior our study. This Bet v 1 isoallergen has sequence identity as little as 65% when compared with other isoallergens and variants (Fig 4). Bet v 1.0301 shares only between 65% to 74% sequence identity with other Bet v 1 isoallergens and variants. The IgE binding properties of this particular sequence variant may very likely be different when, i.e., compared to Bet v 1.0101 or Bet v 1.0201. If one compares the sequence identity of Bet v 1.0301 to food allergens, it varies between 63% and 66% for Mal d 1, Pur av 1, and Fra a 3, and up to 84%-86% similarity to four Cor a 1 sequence variants (Cor a 1.0401, Cor a 1.0402, Cor a 1.0403, and Cor a 1.0404). It is surprising that Bet v 1.0301 shows higher sequence identity to Cor a 1 variants than to any of its Bet v 1 homologues in birch pollen.

It is interesting to note that isoallergens with a similarly low degree of sequence identity were proven to have a different diagnostic specificity and a different performance to detect clinically relevant sensitization [35, 36]. Thus, it could be worthy to study whether this has an impact on IgE cross-reactivity in birch pollen allergic patients also suffering from allergy to hazelnut.

This demonstrates that a comprehensive knowledge about the isoallergen and variant composition is crucial to evaluate the quality of an allergen product. Moreover, we found that the current MS-data base search engines are not yet designed to allow an unambiguous automatic identification of closely related sequence variants. Even though we studied only one vendor specific software tool, we assume that other search engines will show similar limitations.

Our iterative manual data analysis process led to reliable results and allowed explaining the experimental MS data with the lowest possible number of protein entries, rather than reporting either redundant sequences or even false positive hits. However, automated analysis is needed to apply this process to routinely identify sequence variants within research projects, e.g., studying the Bet v 1 isoallergen composition of different *Betula pendula* cultivars. Such studies would provide valuable information about the biological variability of the Bet v 1 isoallergen and variant composition and would allow to assess its impact on the diagnostic or therapeutic allergen products.” This applies also to analyses within a regulatory setup, e.g., for batch control of allergen products. Other software tools may perform differently compared with PLGS, but it seems very unlikely that the scoring of any software tool available to date is not biased by combining peptide scores of commonly shared peptides with scores of isoallergen specific peptides. This means that an individual sequence variant is essentially identified on the basis of only one single spectrum and should accordingly be assigned with a total protein score that indicates a fairly high risk of being a false positive hit instead including also peptide scores for all other commonly shared peptides.

## Supporting Information

S1 File Figure A: Annotated reference 2D maps of birch pollen extract used for protein identification by LC-MS^E^. 50μg of protein extract was run on each 2D-PAGE gel. A) 3–10 pH non-linear gradient; B) 3–6 pH linear gradient; C) 4–7 pH linear gradient gel-1; D) 5–8 pH linear gradient gel-2; E) 3–10 pH linear gradient. F) 4–7 pH linear gradient gel-2. All pH strips were 11cm in length. Spots of interest from SyproRuby stain gels were excised, submitted to tryptic digestion and analyzed by MS^E^. Identified spots are numbered and circled in red. MW–Molecular weight marker. Table A: Summary of the MS data of the identification non-allergenic proteins. Table B: Summary of the MS data of the identification Bet v 2, 4, 6 and 7. Table C: Summary of the MS data of the identification Bet v 1 isoallergens and variants. Table D: Fragmentation spectra referring to the peptide sequences specified in Table C, column P*. (PDF)

## Abbreviations

IUIS: International Union of Immunological Societies
PLGS: ProteinLynx Global Server
