# Supplementary material for: Quality Control of Biomedicinal Allergen Products – Highly Complex Isoallergen Composition Challenges Standard MS Database Search and Requires Manual Data Analyses
Source: PLoS One. 2015 Nov 11;10(11):e0142404. doi: 10.1371/journal.pone.0142404 (PMC4641616; doi:10.1371/journal.pone.0142404)

Table S1: Summary of the MS data of the identification non-allergenic proteins of birch pollen

Gel	Spot	Acc. No.	Protein name	score	SeqCov	P.	Error
S2	4	B9HRM9	Predicted protein Fragment-P.trichocarpa	673	13	2	4.4
	5	D9ZIQ8	BZIP domain class transcription factor	107	2	1	1.6
		D8TBQ5	Putative uncharacterized protein	23	1	1	2.2
	6	B9HRM9	Predicted protein Fragment-P.trichocarpa	1253	16	3	3.0
	8	Q676X7	Serine threonine kinase-H.orientalis	2064	24	3	2.4
	10	Q676X7	Serine threonine kinase-H.orientalis	3645	24	3	3.2
	12	P42739	Polyubiquitin Fragment-A.cliftonii	89	4	2	2.5
	19	B9HRM9	Predicted protein Fragment-P.trichocarpa	342	8	1	4.2
	26	C6TN38	Putative uncharacterized protein	120	5	1	0.5
	29	Q5QNF4	Os01g0217100 protein-O.sativa	261	6	1	9.9
		P42644	14 3 3 like protein-A.thaliana	1121	16	5	2.8
	35	Q7EZT3	Putative GTP binding protein-O.sativa	151	2	1	2.3
	36	A5JTQ2	Beta xylosidase alpha L arabinofuranosidase	828	2	1	3.5
	37	A5JTQ2	Beta xylosidase alpha L arabinofuranosidase	2177	3	3	2.6
	38	A5JTQ2	Beta xylosidase alpha L arabinofuranosidase	994	3	3	2.7
S3	2	P85925	Unknown protein 18 Fragment-P.menziesii	12958	100	2	3.0
		D7R774	Putative glycine rich RNA binding protein	1897	23	3	2.0
		A9PBC3	Glutaredoxin-P.trichocarpa	182	14	1	4.8
		D8T3L5	Putative uncharacterized protein	98	4	1	4.7
	3	A5Z282	Glutathione peroxidase Fragment	646	13	2	1.6
		B5M1Y7	Glutathione peroxidase-R.australe	142	4	1	2.3
	4	B9HRM9	Predicted protein Fragment-P.trichocarpa	5091	21	3	4.0
	10	A8HTK0	Thioredoxin-Glycine max	457	15	2	5.2
	25	P42739	Polyubiquitin Fragment-A.cliftonii	11956	11	6	2.6
S4	18	C5YD96	Putative uncharacterized protein-S.bicolor	5	0	1	10.9
	19	D2X9U2	Lambda class glutathione transferase	574	9	2	7.4
		D2WL71	Lambda class glutathione transferase	342	8	2	8.3
		Q2XNS9	Putative uncharacterized protein-A.officinalis	148	4	1	6.0
	43	Q8LEB8	Quinone oxidoreductase like protein	239	8	3	4.3
	49	A9PIJ2	Predicted protein-P.trichocarpa	141	7	2	5.2
	58	B3VDY8	UTP glucose 1 phosphate uridylyltransferase	195	5	2	1.7

Gel	Spot	Acc. No.	Protein name	score	SeqCov	P.	Error
S5	1	P42739	Polyubiquitin Fragment-A.cliftonii	11310	9	8	2.3
	2	B4FI58	Putative uncharacterized protein-Zea mays	436	3	1	2.8
	3	B4FI58	Putative uncharacterized protein-Zea mays	535	3	1	1.4
		D7KLT8	Putative uncharacterized protein-A.lyrata	46	8	2	5.4
	5	P85925	Unknown protein 18 Fragment-P.menziesii	1784	100	2	1.7
		D7TQB6	Putative uncharacterized protein-V.vinifera	1219	14	5	5.6
		D7M9C8	Putative uncharacterized protein-A.lyrata	260	6	1	6.9
		C4MF37	UDP glycosyltransferase Fragment-A.strigosa	174	11	1	5.5
		Q20CC1	Fgenesh protein 125-B.vulgaris	133	8	1	5.7
		A2ZNJ7	Putative uncharacterized protein-O.sativa	53	3	3	3.9
	6	D1LWT8	Triosephosphate isomerase-D.longan	3028	10	3	3.3
		D7KLT8	Putative uncharacterized protein-A.lyrata	52	8	2	6.9
		E1Z4S1	Putative uncharacterized protein-C.variabilis	47	1	1	1.6
	8	D7TQB6	Putative uncharacterized protein-V.vinifera	1677	13	3	3.6
		Q9FQ95	In2 1 protein-Glycine max	547	5	1	2.5
	10	A5BNR6	Putative uncharacterized protein-Vitis vinifera	884	4	1	0.5
	11	C6T942	Putative uncharacterized protein-Glycine max	1248	4	1	1.0
		C1IC54	Cinnamyl alcohol dehydrogenase-G.hirsutum	233	6	2	1.8
		F2CUL2	Predicted protein-H.vulgare var distichum	214	3	1	2.4
	21	A2ICP9	Ran1-P.sativum	1108	18	5	2.5
		D7KLT8	Putative uncharacterized protein-A.lyrata	97	8	2	3.3
	22	C6T942	Putative uncharacterized protein-Glycine max	160	4	1	1.1
	23	G7K694	Fructose bisphosphate aldolase-M.truncatula	722	4	3	2.4
	24	F6GVW7	Putative uncharacterized protein-V.vinifera	386	14	4	2.9
	27	B0FGG5	Monodehydroascorbate reductase	1222	3	1	1.4
	28	D2D2Z8	Phosphoglycerate kinase-G.hirsutum	194	11	3	4.2
	30	B5SVF6	Alcohol dehydrogenase Fragment-D.arborea	1396	10	2	3.3
S1	4	B9HRM9	Predicted protein Fragment-P.trichocarpa	837	8	1	3.2
		B9REC4	Serine threonine protein kinase putative	157	1	2	7.8
		D8UG87	Putative uncharacterized protein-V.carteri	101	4	1	1.1
	5	B9HRM9	Predicted protein Fragment-P.trichocarpa	2045	23	3	3.7
	7	O49813	Olee1 like protein-B.pendula	913	46	11	4.4

Gel & Spot refer to supplemantary figure 1; Acc.No. refers to UniProt; SeqCov gives sequence coverage in %; P is number of identified peptides; Error is RMS mass error in ppm.

Gel	Spot	Acc. No.	Allergen	Score	SeqCov	P	Error
B	2	A4K9Z8	Bet v 2	2802	17	2	2.5
	3	Q39419	Bet v 4	558	26	2	2.0
	7	A4K9Z8	Bet v 2	4268	8	1	2.0
	9	A4K9Z8	Bet v 2	1079	8	1	4.1
	16	A4K9Z8	Bet v 2	11242	57	8	3.4
D	9	Q9FUW6	Bet v 6	35265	75	23	3.5
	12	Q9FUW6	Bet v 6	17583	43	14	2.7
	31	Q9FUW6	Bet v 6	3350	22	8	3.0
E	2	Q8L5T1	Bet v 7	291	28	4	1.6
	8	Q8L5T1	Bet v 7	1073	39	7	3.2
	9	Q8L5T1	Bet v 7	1140	47	7	7.2
	10	Q8L5T1	Bet v 7	626	35	6	7.7
	11	Q8L5T1	Bet v 7	605	35	6	8.1

Gel & Spot refer to figure 1; Acc.No. refers to UniProt; SeqCov gives sequence coverage in %; P is number of identified peptides; Error is RMS mass error in ppm

Table S2: Summary of the MS data of the identification Bet v 2, 4, 6 and 7

Table S3: Summary of the MS data of the identification Bet v 1 isoforms and variants

Gel	Spot	UniProt	Seq. Cov.%	assigned peptides	Protein Error	N	P*	Score	Peptide Error
B	20	Q9SCI0	74	T1,T2,T3,T4,T6,T7,T8,T10,T11	5.2	4	-	-	-
		Q39427	49	T1,T2,T3,T8,T11	4.6		-	-	-
		P43177	47	T2,T3,T7,T8,T10,T11	5.1		T2: AFILDGDNLVPK	8.2	0.9
		P43180	47	T2,T3,T7,T8,T10,T11	5.1		T2: AFILEGDNLIPK	7.5	2.8
C	5	P43186	55	T1,T2,T3,T4,T8,T11	2.0	2	-	-	-
		P15494	41	T3,T6,T8,T11	2.0		-	-	-
	6	Q9SCI0	76	T1,T2,T3,T4,T6,T7,T8,T10,T11	3.6	5	-	-	-
		P43180	68	T1,T2,T3,T6,T7,T8,T10,T11	4.0		T2: AFILEGDNLIPK	8.7	0.6
		P43186	60	T1,T2,T3,T4,T8,T10,T11	4.3		T2: AFILDGDNLIPK	9.0	1.0
		Q96370	74	T1,T2,T3,T4,T6,T7,T8,T10,T11	3.8		-	-	-
		O23748	75	T1,T2,T3,T4,T6,T7,T8,T10,T11	3.8		-	-	-
	7	P15494	31	T3,T8,T11	2.3	1	-	-	-
	8	P43177	67	T1,T2,T3,T6,T7,T8,T10,T11	3.9	5	-	-	-
		P43180	67	T1,T2,T3,T6,T7,T8,T10,T11	3.1		T2: AFILEGDNLIPK	8.2	-1.8
		P43179	67	T1,T2,T3,T6,T7,T8,T10,T11	3.2		-	-	-
		P15494	56	T2,T3,T6,T7,T8,T10,T11	2.8		-	-	-
		P43186	55	T1,T2,T3,T4,T8,T11	2.7		T2: AFILDGDNLIPK	8.0	-1.8
	11	P43177	78	T1,T2,T3,T5,T6,T7,T8,T9,T10,T11	6.1	7	T1 MGVFNYEIETTSVIPAAR	9.0	2.1
		P43180	78	T1,T2,T3,T5,T6,T7,T8,T9,T10,T11	6.1		-	-	-
		P43186	74	T1,T2,T3,T4,T6,T8,T11	8.1		-	-	-
		P15494	84	T1,T2,T3,T4,T5,T6,T7,T8,T9,T10,T11	6.1		-	-	-
		Q96370	84	T1,T2,T3,T4,T5,T6,T7,T8,T9,T10,T11	6.4		T6: YNYSVIEGGPMGDTLEK	9.8	1.0
		P43178	84	T1,T2,T3,T4,T5,T6,T7,T8,T9,T10,T11	3.3		-	-	-
		O23748	78	T1,T2,T3,T4,T6,T7,T8;T9,T10,T11	6.5		T6: YSYSLIEGGPVGDTLEK	8.2	2.0

Gel	Spot	UniProt	Seq. Cov.%	assigned peptides	Protein Error	N	P*	Score	Peptide Error
C	12	P43186	34	T1,T2,T8,T11	2.9	2	-	-	-
		P15494	24	T2,T8,T11	2.6		-	-	-
	15	P15494	49	T1,T2,T3,T8,T11	2.7	1	-	-	-
	16	P43186	79	T1,T2,T3,T4,T5,T6,T7,T8,T10,T11	3.0	3	-	-	-
		P43180	41	T1,T2,T3,T11	3.2		-	-	-
		P15494	66	T1,T2,T3,T4,T6,T8,T11	2.9		-	-	-
	17	Q39427	40	T1,T2,T3,T8	2.6	2	T2: AFILDGDNLIPK	6.6	0.7
		Q96370	40	T1,T2,T3,T8	2.1		T2: AFILDGDNLVPK	6.7	-0.5
	18	P43186	70	T2,T3,T4,T5,T6,T7,T8,T10,T11	5.1	4	-	-	-
		P15494	72	T1,T2,T3,T4,T5,T6,T8,T11	6.6		-	-	-
		P43180	37	T2,T3,T5,T11	5.9		-	-	-
		O23749	69	T1,T2,T3,T4,T5,T6,T8,T10,T11	5.1		T10: EKGETLLR	8.0	1.6
	19	Q39431	67	T1,T2,T3,T4,T6,T7,T10,T11	4.8	5	T6: YSYSVIEGGPIGDTLEK	7.6	2.5
		P43177	84	T1,T2,T3,T4,T5,T6,T7,T8,T9,T10,T11	5.0		-	-	-
		P43180	73	T2,T3,T4,T5,T6,T7,T8,T9,T10,T11	5.2		-	-	-
		Q0QLS9	71	T2,T3,T4,T5,T6,T8,T9,T10	4.6		T8: IVATPDGGCILK	8.0	4.1
		Q39427	49	T1,T2,T3,T8,T11	5.0		-	-	-
	20	P43177	81	T1,T2,T3,T4,T5,T6,T7,T8,T10,T11	3.7	5	-	-	-
		P43183	81	T1,T2,T3,T4,T5,T6,T7,T8,T10,T11	3.6		-	-	-
		P43179	81	T1,T2,T3,T4,T5,T6,T7,T8,T10,T11	3.6		-	-	-
		P43180	81	T1,T2,T3,T4,T5,T6,T7,T8,T10,T11	3.8		-	-	-
		Q9SCH6	68	T1,T2,T3,T4,T5,T6,T7,T11	3.9		-	-	-
	21	P43184	81	T1,T2,T3,T4,T5,T6,T7,T8,T10,T11	4.1	4	T2: AFILEGDTLIPK	9.8	-11.7
		P43180	64	T1,T2,T3,T6,T8,T10,T11	4.2		-	-	-
		P15494	54	T2,T3,T6,T8,T10,T11	4.5		-	-	-
		P43177	64	T1,T2,T3,T6,T8,T10,T11	4.6		-	-	-

Gel	Spot	UniProt	Seq. Cov.%	assigned peptides	Protein Error	N	P*	Score	Peptide Error
	24	P43177	49	T2,T3,T6,T8,T11	2.8	2	-	-	-
		P43179	34	T3,T6,T11	3.1		-	-	-
	26	P43177	72	T1,T2,T3,T4,T5,T6,T8,T11	3.0	2	-	-	-
		P15494	58	T1,T3,T5,T6,T8,T11	3.4		-	-	-
	27	O23749	73	T1,T2,T3,T4,T6,T7,T8,T10,T11	4.0	2	T2: AFILDGDNLIPK	9.2	2.6
		P43180	73	T1,T2,T3,T4,T6,T7,T8,T10,T11	4.7		T2: AFILEGDNLIPK	8.1	2.3
	28	O23749	88	T1,T2,T3,T4,T5,T6,T7,T8,T9,T10,T11	4.7	3	-	-	-
		Q0QLV2	83	T1,T2,T3,T4,T5,T6,T7,T8,T9,T10	4.9		-	-	-
		Q39415	46	T1,T2,T3,T5,T8	5.8		-	-	-
	29	O23749	74	T1,T2,T3,T4,T6,T7,T8,T10,T11	3.7	2	-	-	-
		Q0QLV2	67	T2,T3,T4,T6,T7,T8,T10	3.5		-	-	-
	30	O23749	50	T1,T2,T3,T4,T8,T10	3.8	2	-	-	-
		P43180	33	T1,T2,T3	3.1		-	-	-
	31	P43177	81	T1,T2,T3,T4,T5,T6,T7,T8,T10,T11	3.4	2	-	-	-
		P43179	56	T1,T3,T5,T7,T8,T10,T11	5.1		-	-	-
	35	P43186	32	T1,T2,T4,T8	3.5	1	-	-	-
	36	P43180	41	T1,T2,T3,T11	2.7	2	-	-	-
		P43186	69	T1,T2,T3,T4,T6,T7,T8,T11	3.3		-	-	-
	37	P43180	41	T1,T2,T3,T11	4.5	2	-	-	-
		P43186	55	T1,T2,T3,T4,T8,T11	3.1		-	-	-

Gel & Spot: nomenclature refer to figure 1; **Seq Cov.**: sequence coverage was manually calculated including only pass one matches; **T:** peptide nomenclature refers to figure 3; **protein error:** precursor RMS mass error in ppm; **N:** number of isoforms per spot; **P:** aminoacid sequence of peptides if protein was identified with one peptide only; ***:** fragmentation spectra of these peptides are shown in supplementary table S4 **Score:** protein score as by PLGS; **peptide error**: individual precursor mass error in ppm

Gel 3-6pH	Acc. No.
Spot 20	P43177
PLGS peptide score	8.2
Peptide MH+Error(ppm)	0.9

Table S4: Fragmentation spectra refering to the peptide sequences specified in table S3, column P*

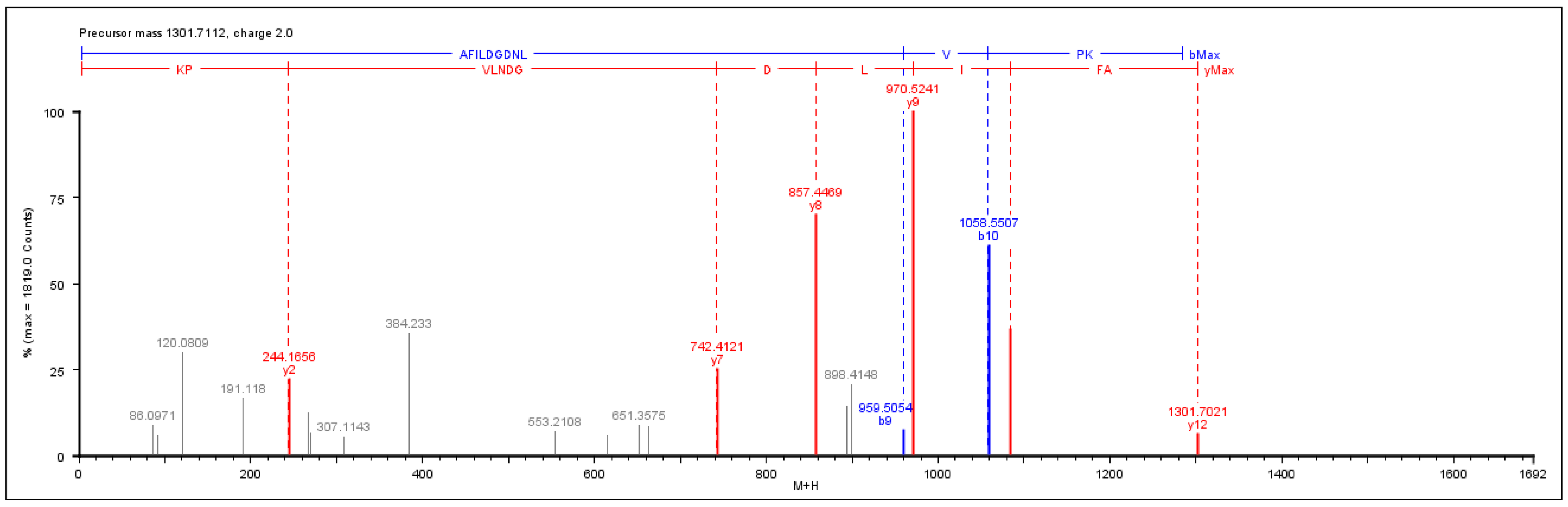

	P43180
PLGS peptide score	7.5
Peptide MH+Error(ppm)	2.8

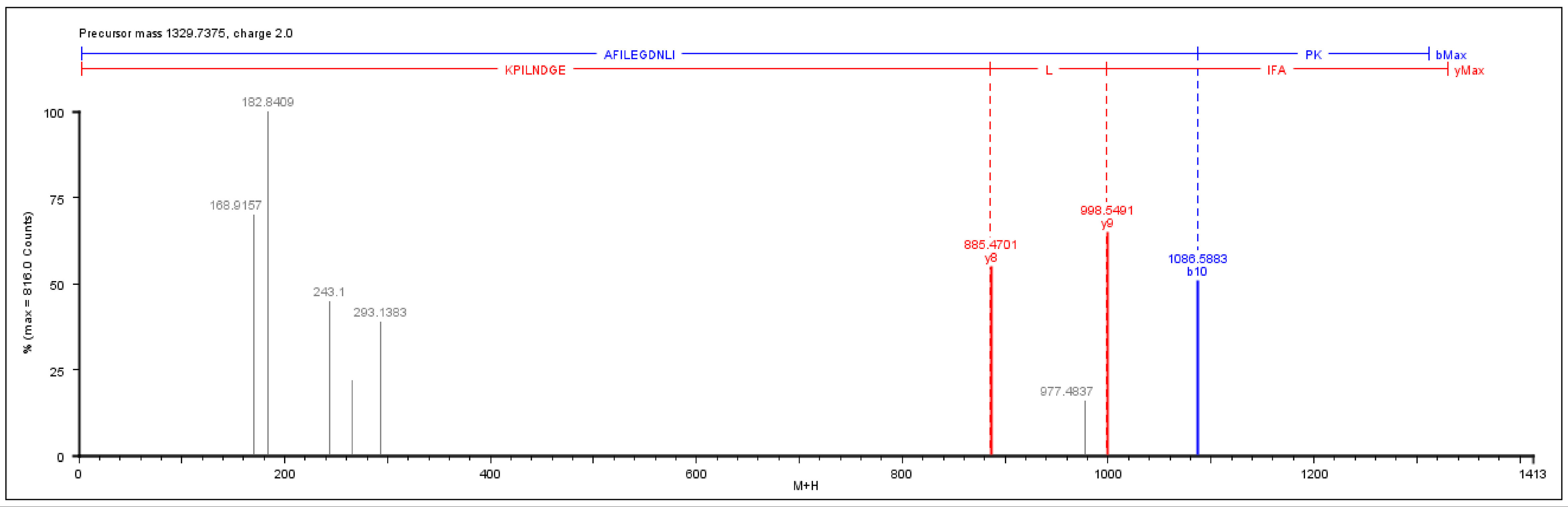

Gel 4-7pH	Acc. No.
spot 6	P43180
PLGS peptide score	8.7
Peptide MH+Error(ppm)	0.6

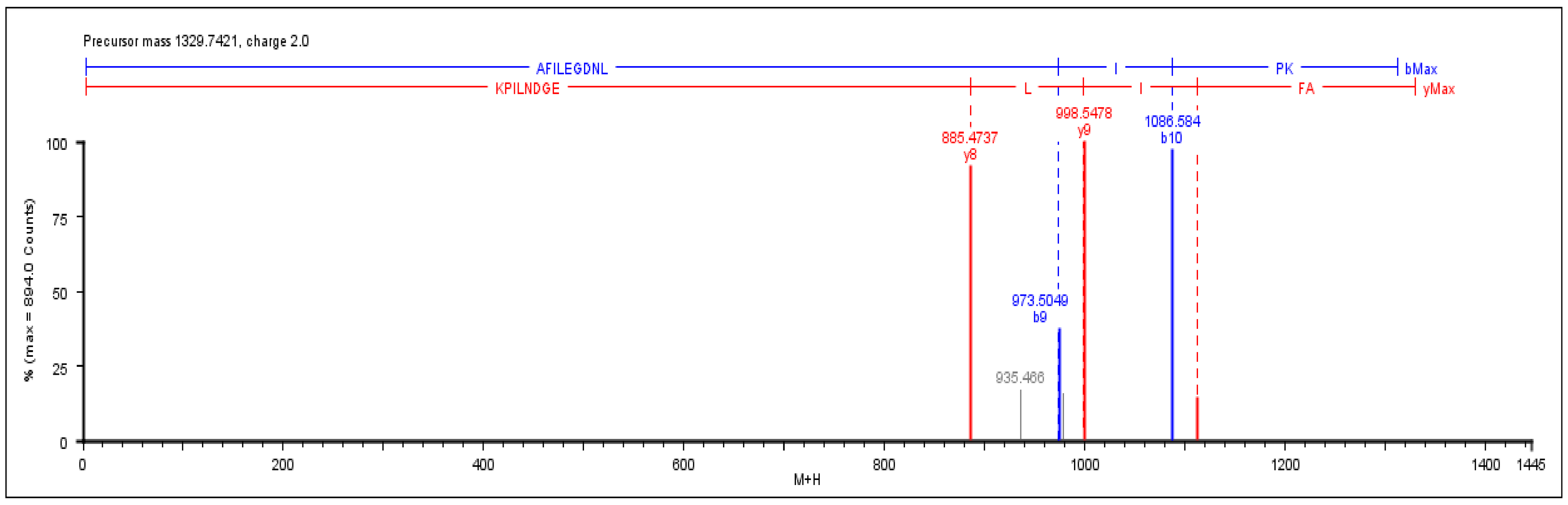

	P43186
PLGS peptide score	9.0
Peptide MH+Error(ppm)	1.0

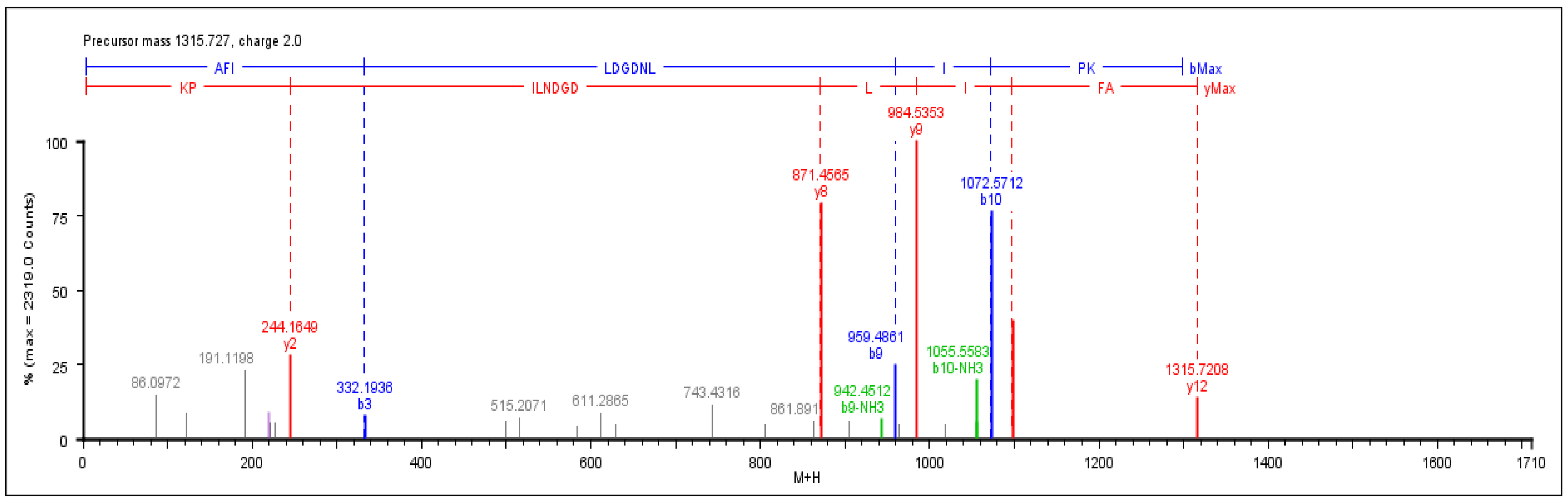

spot 8	P43180
PLGS peptide score	8.2
Peptide MH+Error(ppm)	-1.8

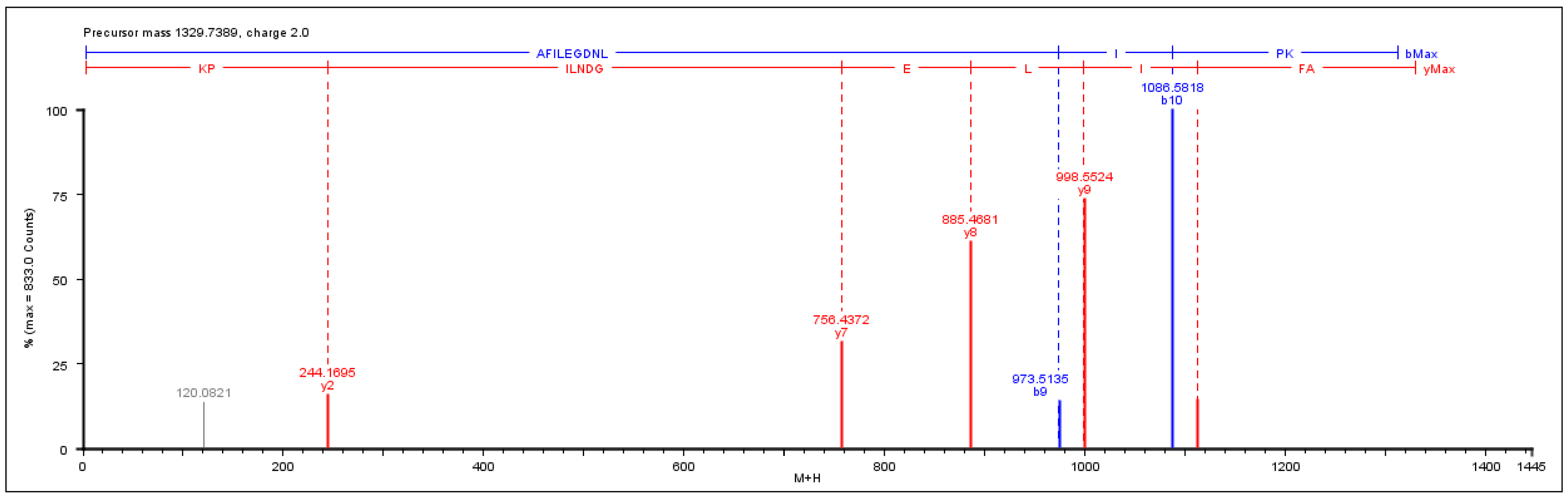

	P43186
PLGS peptide score	8.0
Peptide MH+Error(ppm)	-1.8

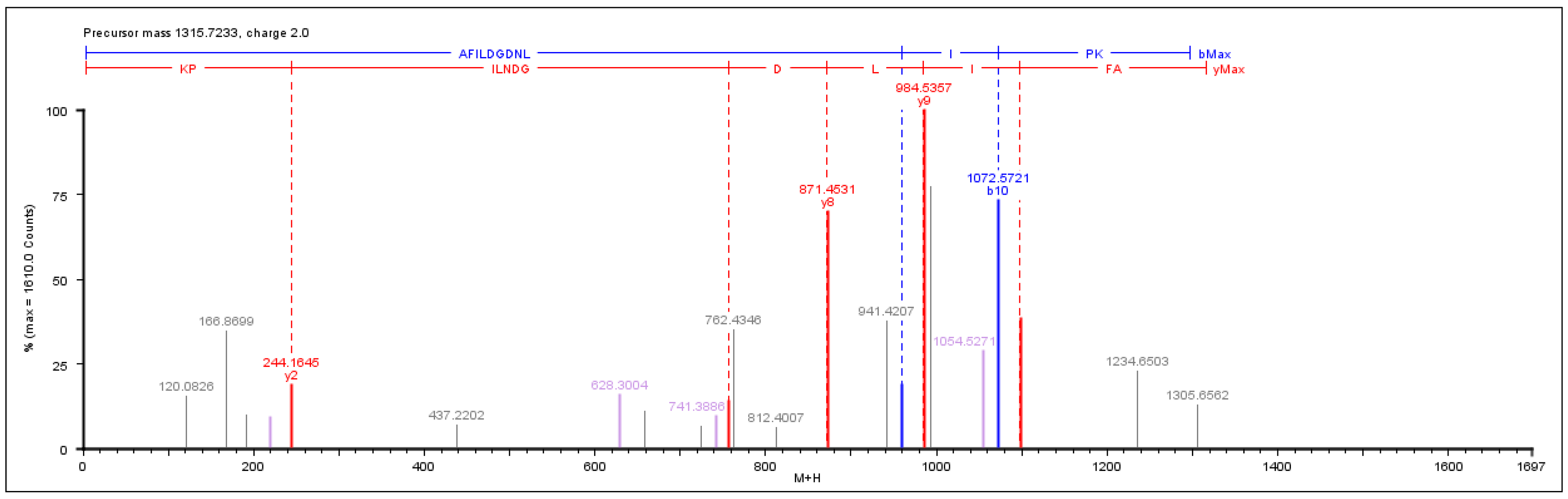

spot 11

P43177

PLGS peptide score 9.0

Peptide MH+Error(ppm) 2.1

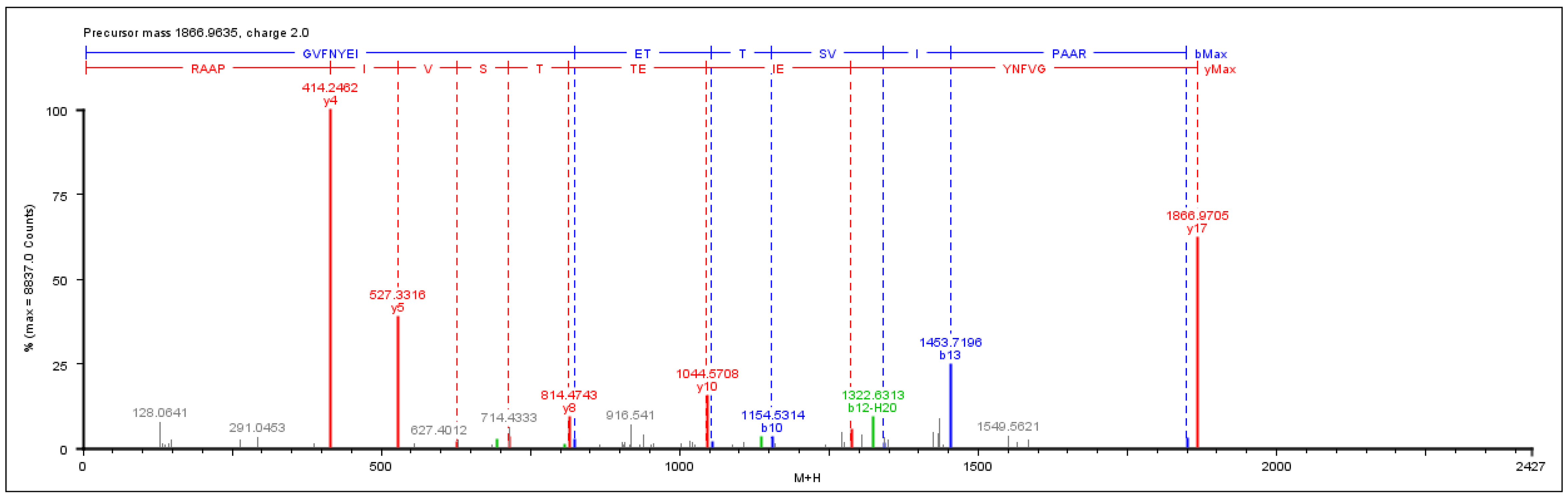

Q96370

PLGS peptide score 9.8

Peptide MH+Error(ppm) 1.0

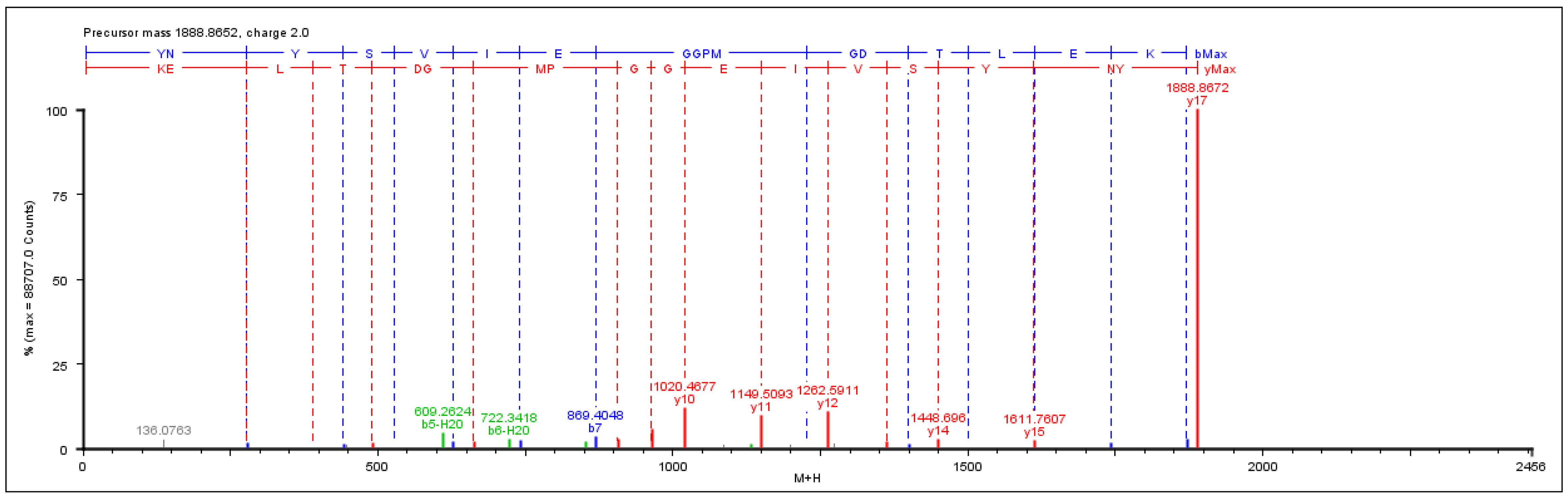

O23748

PLGS peptide score 8.2

Peptide MH+Error(ppm) 2.0

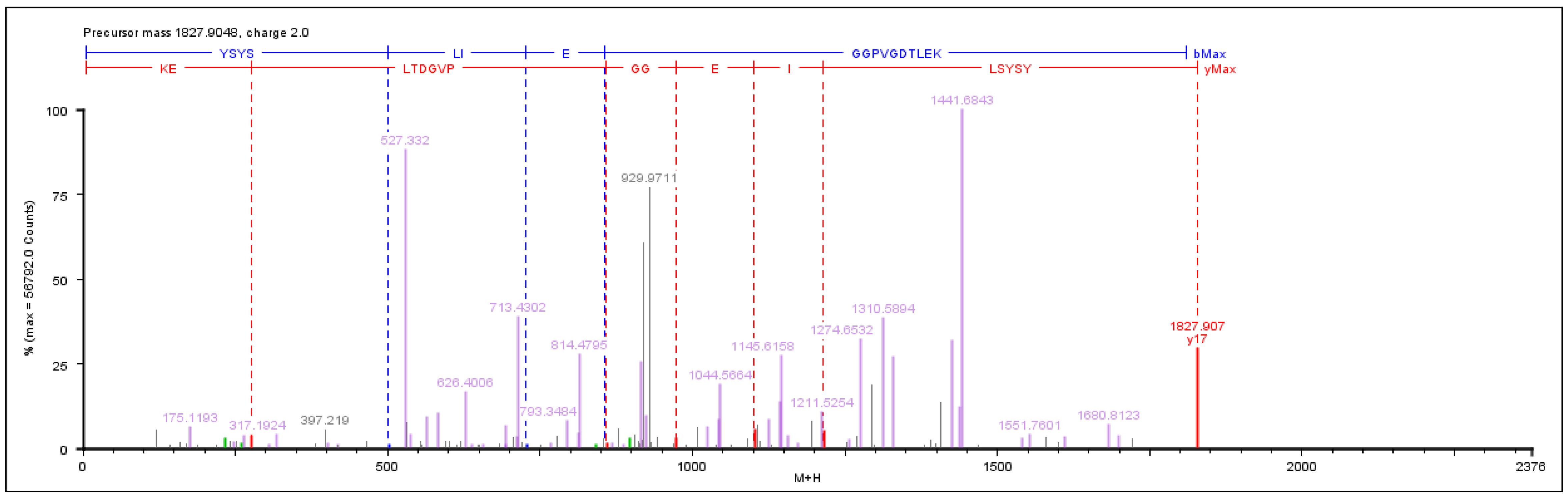

Spot 17

Q39427

PLGS peptide score 6.6

Peptide MH+Error(ppm) 0.7

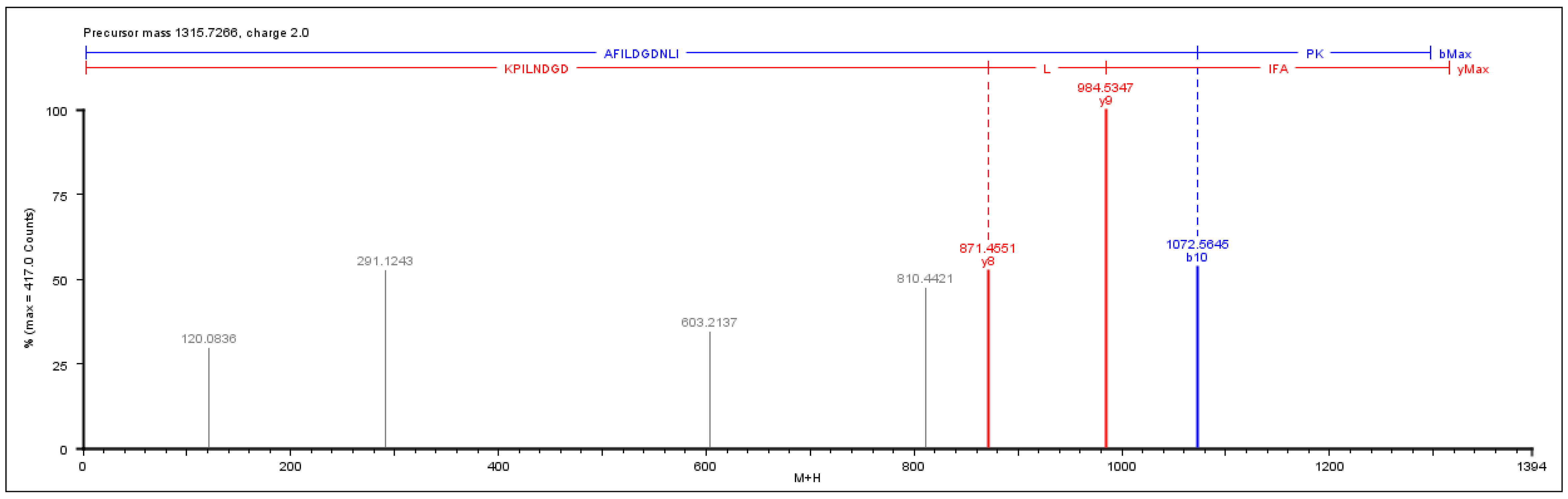

Q96370

PLGS peptide score 6.7

Peptide MH+Error(ppm) -0.5

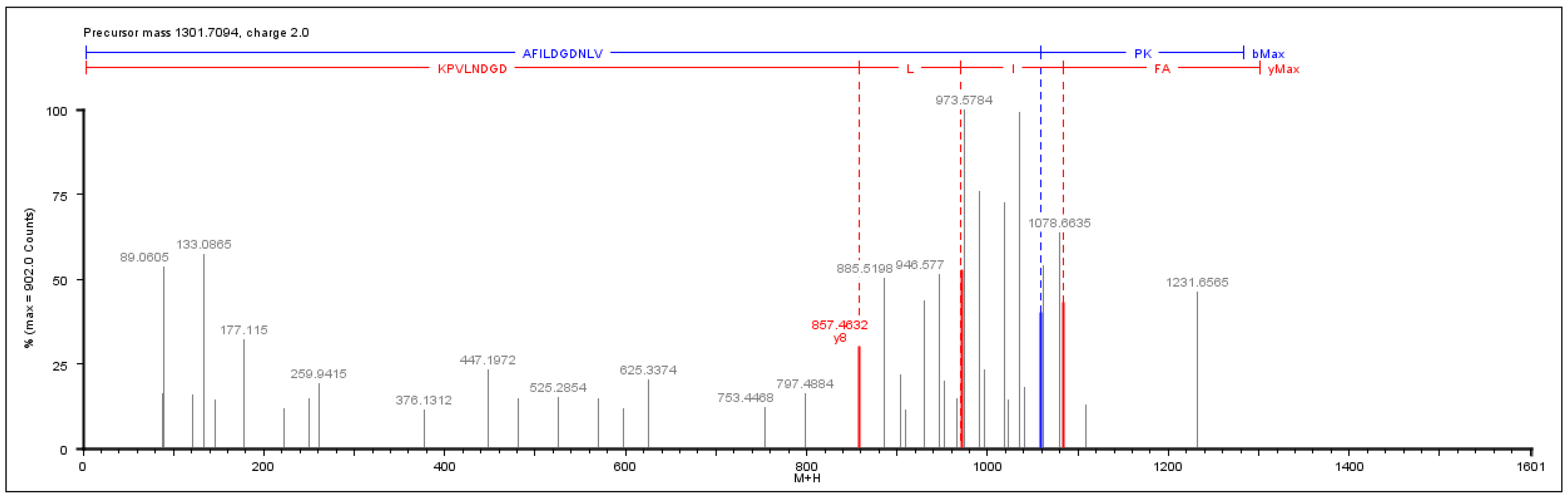

---

spot 18

O23749

PLGS peptide score 8.0

Peptide MH+Error(ppm) 1.6

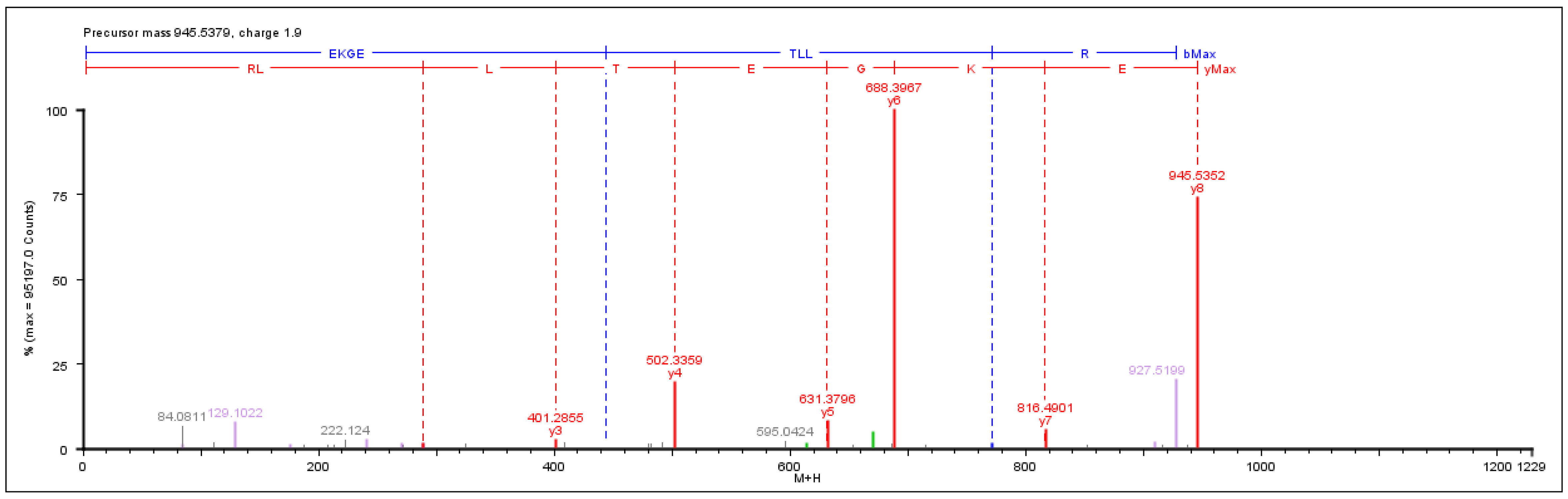

---

spot 19 Q0QLS9

PLGS peptide score 8.0

Peptide MH+Error(ppm) 4.1

Missing spectra for q39427

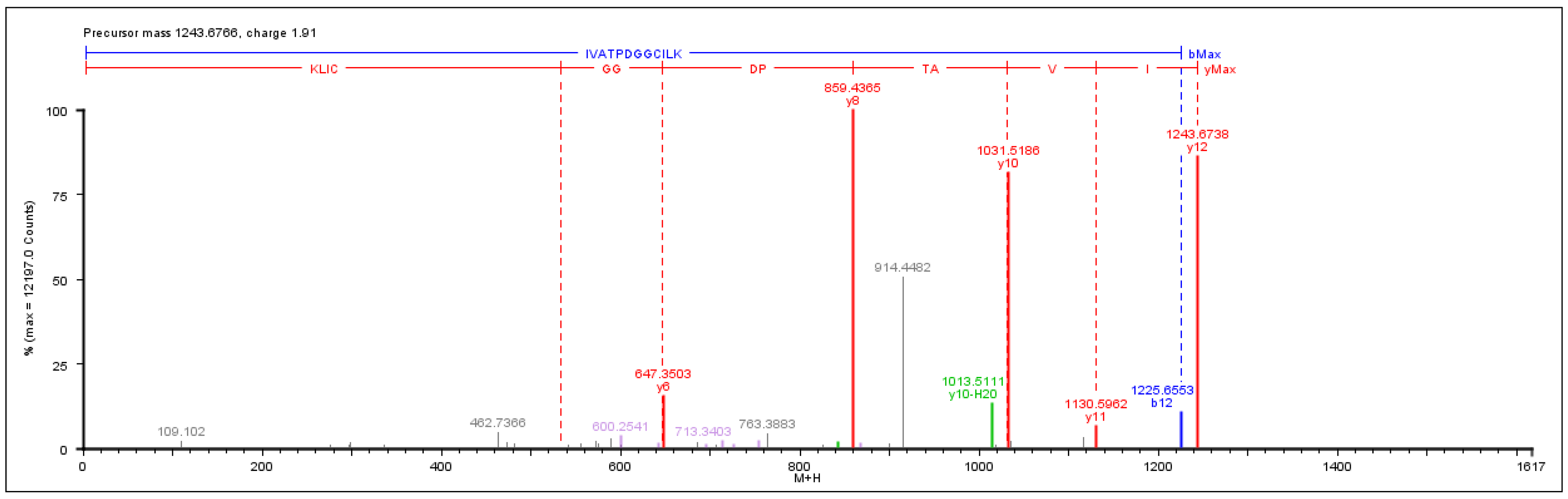

Q39431

PLGS peptide score 7.6

Peptide MH+Error(ppm) 2.5

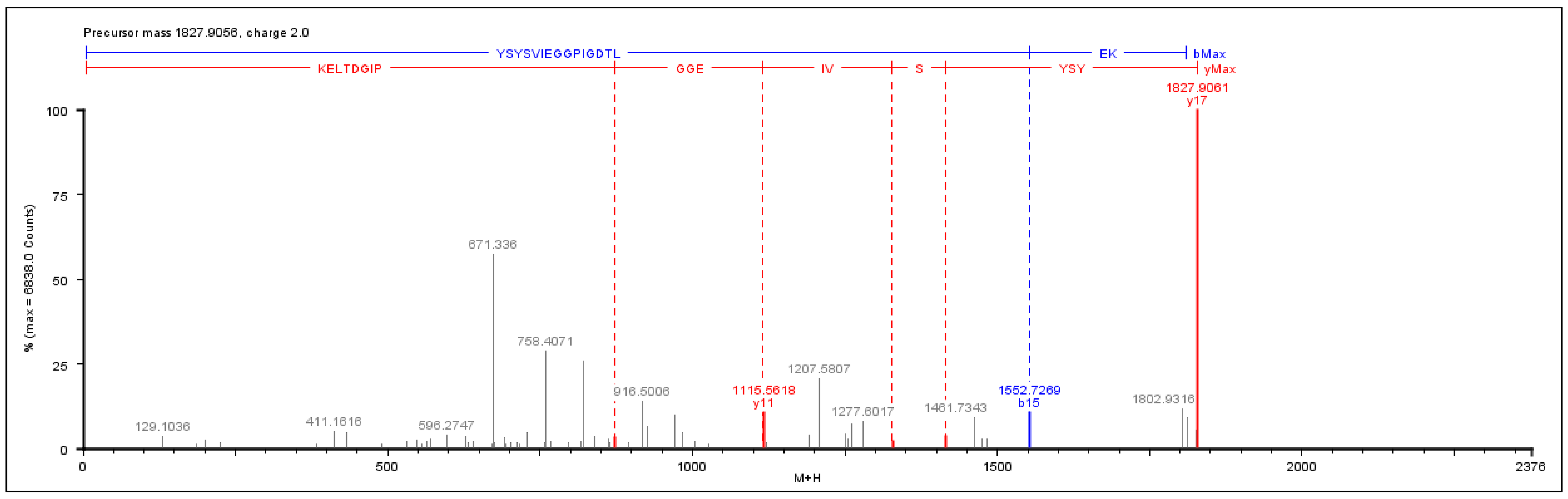

Spot 21 P43184

PLGS peptide score 9.8

Peptide MH+Error(ppm) -11.7

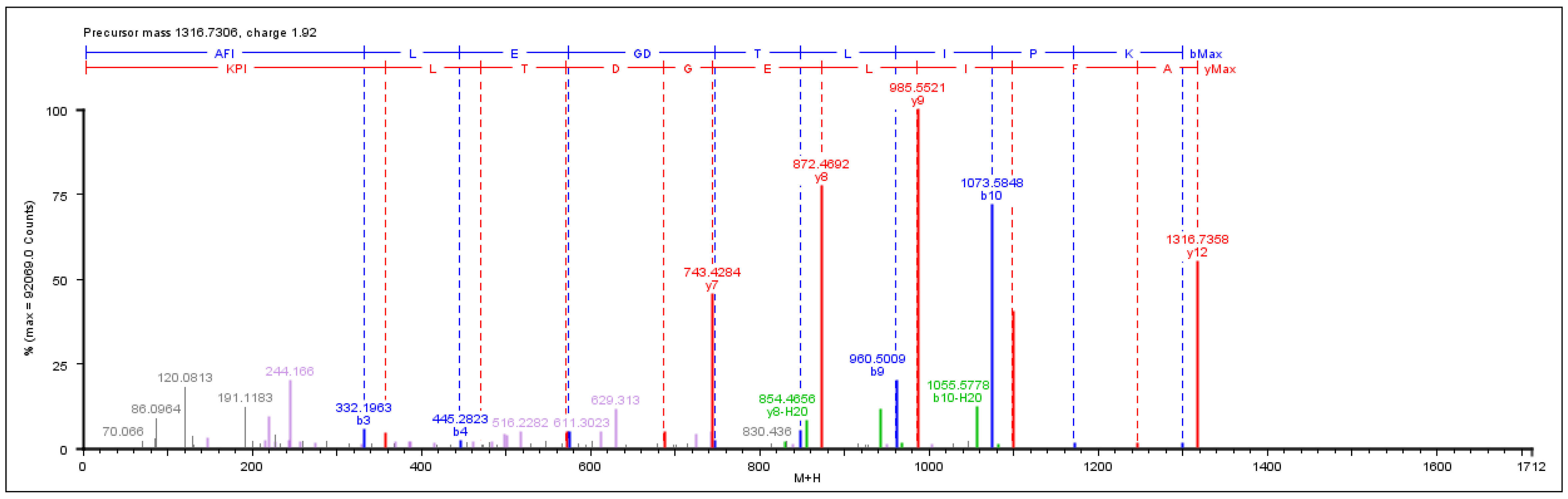

---

spot 27 O23749

PLGS peptide score 9.2
Peptide MH+Error(ppm) 2.6

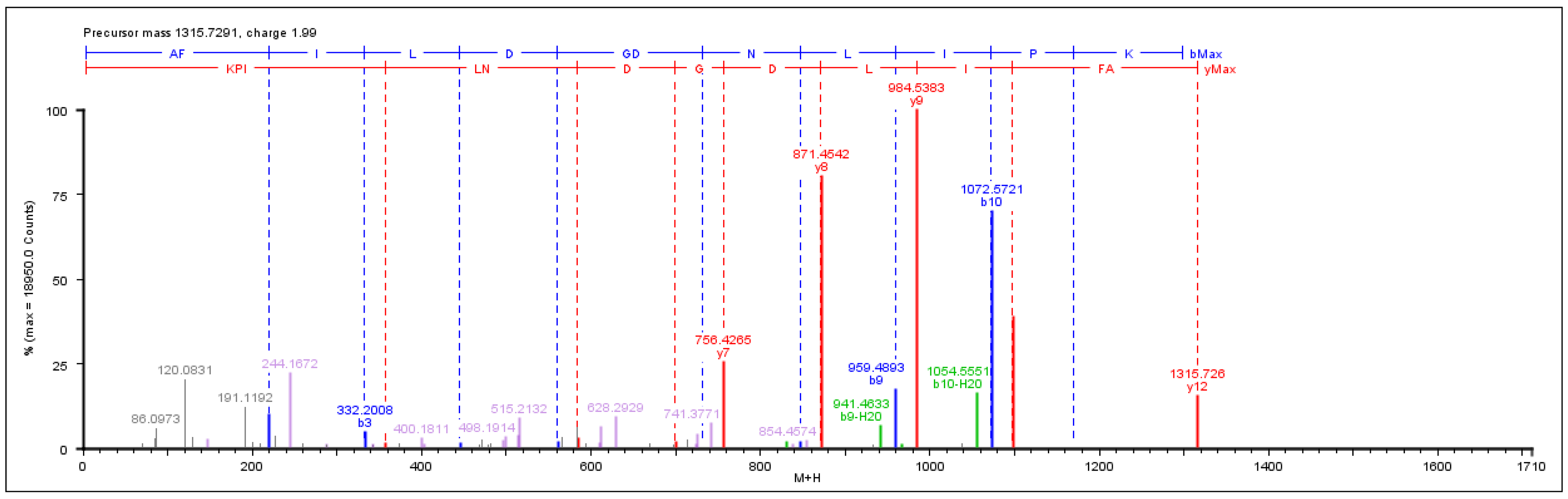

P43180

PLGS peptide score 8.1
Peptide MH+Error(ppm) 2.3

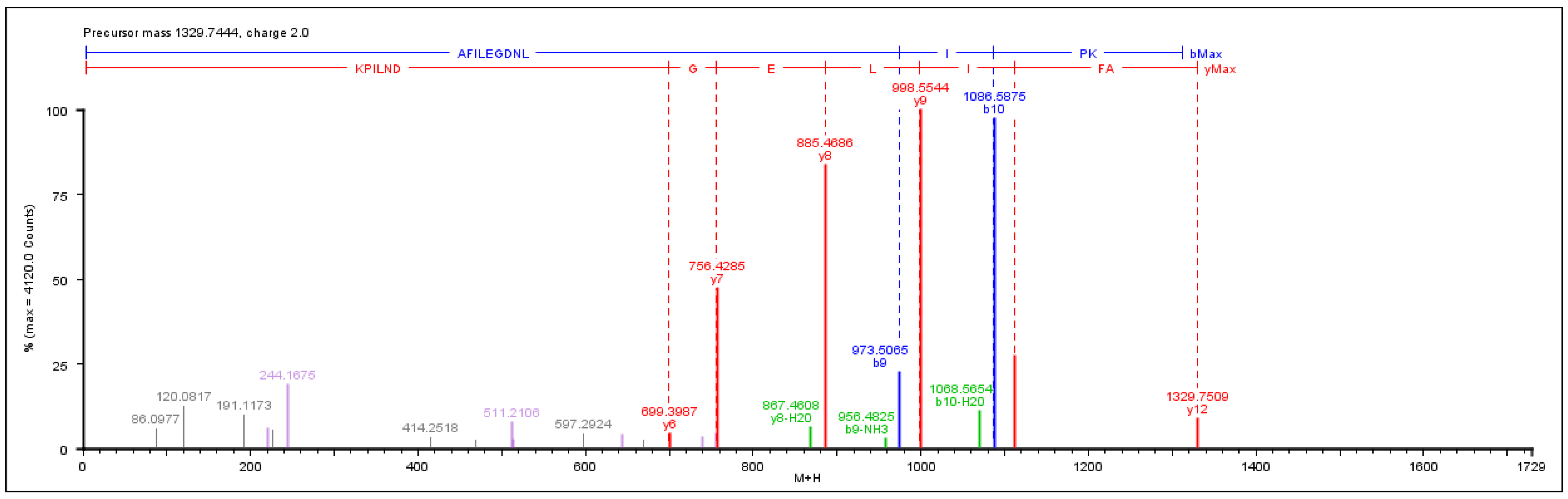

Supplement: S1 File — (PDF) [file pone.0142404.s001.pdf]
